# Supplementary material for: ddcP, pstB, and excess D-lactate impact synergism between vancomycin and chlorhexidine against Enterococcus faecium 1,231,410
Source: PLoS One. 2021 Apr 8;16(4):e0249631. doi: 10.1371/journal.pone.0249631 (PMC8031426; doi:10.1371/journal.pone.0249631)
Supplement: S1 Fig — Representative optical density (OD600) of (A) E. faecium 410 wild-type, (B) ΔpbpZ, (C) ΔvanY, (D) ΔponA, (E) ΔddcP ΔvanY, (F) Δldtfm, (G) ΔddcP Δldtfm, (H) ΔpbpF, and (I) ΔpbpA after vancomycin and chlorhexidine treatment. E. faecium was cultured at 37°C in BHI broth until the OD600 reached 0.6 as described in the materials and methods. Equal volumes of culture were split into BHI containing 0X (control; red circles) or vancomycin and chlorhexidine (Van and H-CHG; blue squares). OD600 values were monitored for 6 h. Error bars indicate standard deviations from three independent experiments. (PDF) [file pone.0249631.s001.pdf]

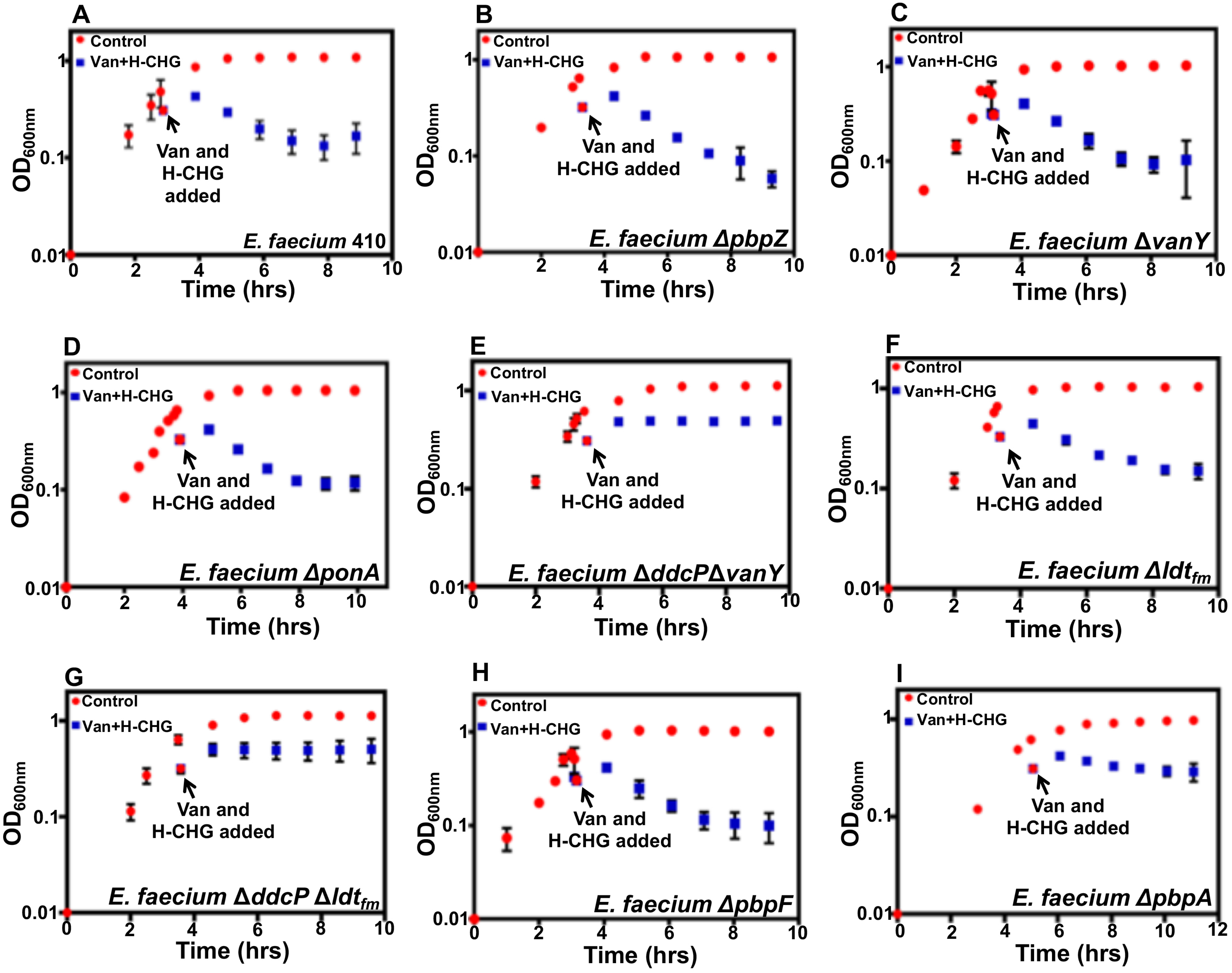

S1 Fig. Representative optical density (OD_{600}) of (A) *E. faecium* 410 wild-type, (B) Δ*pbpZ*, (C) Δ*vanY*, (D) Δ*ponA*, (E) Δ*ddcP* Δ *vanY*, (F) Δ*ldtfm*, (G) Δ*ddcP* Δ*ldt$_{fm}$*, (H) Δ*pbpF*, and (I) Δ*pbpA* after vancomycin and chlorhexidine treatment. *E. faecium* culture was grown at 37°C in BHI until OD_{600} reached 0.6 as described in materials and methods. Equal volumes of cultures were split into BHI containing 0X (control; red circles) or vancomycin and chlorhexidine (Van and H-CHG; blue squares). OD_{600} values were monitored for 6 hr. Error bars indicate standard deviations from n=3 independent experiments.
