# Supplementary material for: ddcP, pstB, and excess D-lactate impact synergism between vancomycin and chlorhexidine against Enterococcus faecium 1,231,410
Source: PLoS One. 2021 Apr 8;16(4):e0249631. doi: 10.1371/journal.pone.0249631 (PMC8031426; doi:10.1371/journal.pone.0249631)
Supplement: S2 Fig — Optical density (OD600nm) (A) and CFU/mL (B) of E. faecium 1,231,410 wild-type (E. faecium 410) and the ddcP deletion mutant with (“treated”) and without (“control”) vancomycin and H-CHG treatment. E. faecium was cultured in BHI broth until the OD600 reached 0.6, as described in materials and methods. Equal volumes of cultures were split into BHI or BHI containing vancomycin (50 μg/ml) and H-CHG (4.9 μg/ml). OD600 values and CFU/mL were monitored for 3 h. Error bars indicate standard deviations from n = 3 independent experiments. Significance was assessed using the one-tailed Student’s t-test. * denotes P-value < 0.05. Stars indicate significant differences between vancomycin- and H-CHG-treated cultures. (PDF) [file pone.0249631.s002.pdf]

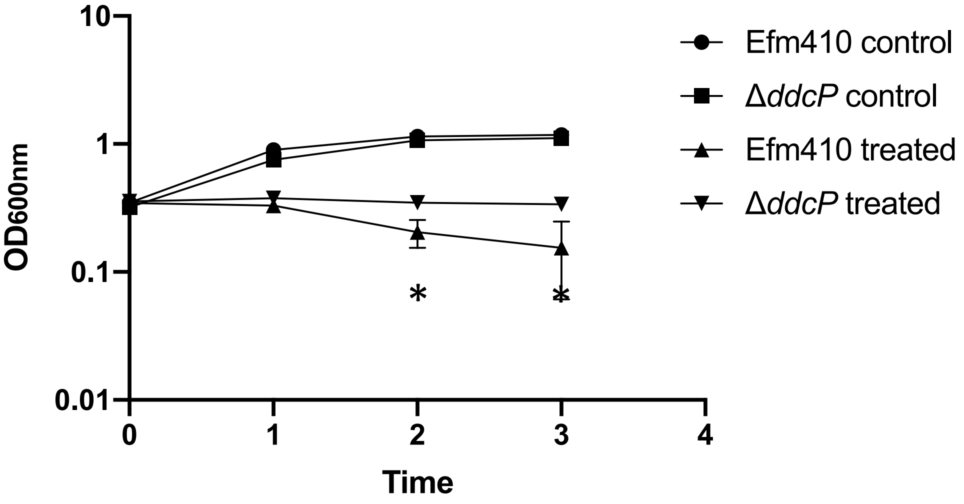

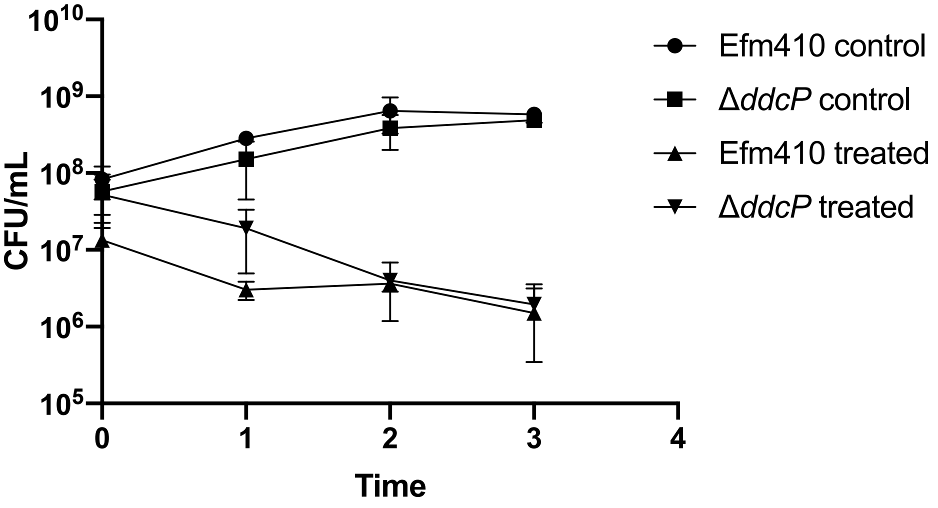

S2 Fig. A Δ*ddcP* mutant dies but does not lyse in the presence of vancomycin and H-CHG. Optical density (OD_{600nm}) (A) and CFU/mL (B) of *E. faecium* 1,231,410 wild-type (*E. faecium* 410) and the *ddcP* deletion mutant with ("treated") and without ("control") vancomycin and H-CHG treatment. *E. faecium* was cultured in BHI broth until the OD_{600} reached 0.6, as described in materials and methods. Equal volumes of cultures were split into BHI or BHI containing vancomycin (50 µg/ml) and H-CHG (4.9 µg/ml). OD_{600} values and CFU/mL were monitored for 3 h. Error bars indicate standard deviations from n=3 independent experiments. Significance was assessed using the one-tailed Student's *t*-test. * denotes *P*-value < 0.05. Stars indicate significant differences between vancomycin- and H-CHG-treated cultures.
