# Supplementary material for: ddcP, pstB, and excess D-lactate impact synergism between vancomycin and chlorhexidine against Enterococcus faecium 1,231,410
Source: PLoS One. 2021 Apr 8;16(4):e0249631. doi: 10.1371/journal.pone.0249631 (PMC8031426; doi:10.1371/journal.pone.0249631)
Supplement: S3 Fig — Intracellular Pi levels were measured for both strains at different growth time points (OD600 0.4–1.0) as described in materials and methods. The levels (pmoles) were normalized using CFU count. Standard deviation was calculated from n = 5 independent experiments and significance value was calculated using one-tailed Student’s t test. Time points: 1, OD600 0.4–0.5; 2, OD600 0.6–0.7; 3, OD600 0.7–0.8; 4, OD600 0.8–0.9; OD600 1.0–1.5. (PDF) [file pone.0249631.s003.pdf]

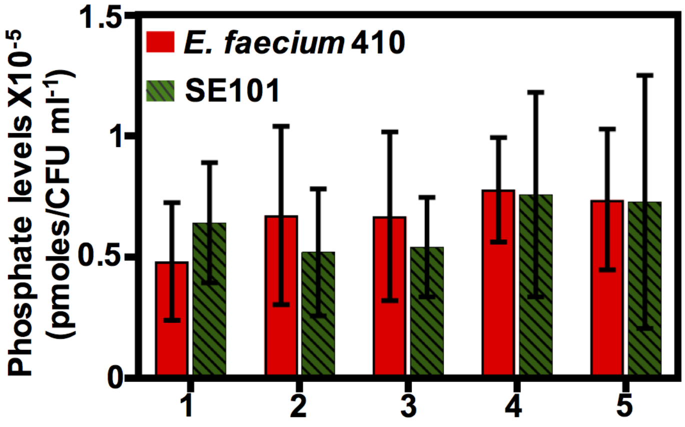

S3 Fig. Quantification of intracellular organic phosphate (Pi) levels in *E. faecium* 410 wild-type and SE101 synergy escaper mutant. Intracellular Pi levels were measured for both strains at different growth time points (OD_{600} 0.4-1.0) as described in materials and methods. The levels (pmoles) were normalized using CFU count. Standard deviation was calculated from n=5 independent experiments and significance value was calculated using one-tailed Student's *t* test. Time points: 1, OD_{600} 0.4-0.5; 2, OD_{600} 0.6-0.7; 3, OD_{600} 0.7-0.8; 4, OD_{600} 0.8-0.9; OD_{600} 1.0-1.5.
