# Supplementary material for: ddcP, pstB, and excess D-lactate impact synergism between vancomycin and chlorhexidine against Enterococcus faecium 1,231,410
Source: PLoS One. 2021 Apr 8;16(4):e0249631. doi: 10.1371/journal.pone.0249631 (PMC8031426; doi:10.1371/journal.pone.0249631)
Supplement: S1 Table — (PDF) [file pone.0249631.s004.pdf]

S1 Table . Primers used in the study

Primer name	Sequence
***pbpF* deletion**	
pbpF flanking Arm1_BamHI For	ATGCAT**GGATCC**ATGGAGCATCAGTCGTTCAGCTGT
pbpF flanking Arm1_Sph1 Rev	ATGCAT**GCATGC**AAAATCCATTTGCTTCTTCTCCTG
pbpF flanking Arm2_Sph1 For	ATGCAT**GCATGC**GGAGAGTAGCCCCTTTGAAAACGA
pbpF flanking Arm2_BamHI Rev	ATGCAT**GGATCC**GGCAACAAGCTGTCTGGTGGCCAA
pbpF screening For	GCAGGAAGAAAACACTCTTTCACT
pbpF screening Rev	CGTTCGATTTGATTCGCGCT
ddcP deletion	
ddcP flanking Arm1_EcoRI For	ATGCAT**GAATTC**CGCCACCTGTCAGTTCAGATCCCA
ddcP flanking Arm1_Sph1 Rev	ATGCAT**GCATGC**TATGGACATATCCGATCTCCTTGT
ddcP flanking Arm2_Sph1 For	ATGCAT**GCATGC**TTGTTTTAATTGAACAAAAAGAAA
ddcP flanking Arm2_EcoRI Rev	ATGCAT**GAATTC**TTGTTCCATGGCATATTGTTTCAT
ddcP screening For	AAACGACCGCTAACCTTCCA
ddcP screening Rev	CGCAAACGTCCATGTACTGC
***vanY* deletion**	
vanY flanking Arm1_BamHI For	ATGCAT**GGATCC**CTAAATATGCCACTTGGGATA
vanY flanking Arm1_Sph1 Rev	ATGCAT**GCATGC**CTTCTTCATTTTCAGTCTCCT
vanY flanking Arm2_Sph1 For	ATGCAT**GCATGC**AGGAGGTAAGGATGGCGGAAT
vanY flanking Arm2_BamHI Rev	ATGCAT**GGATCC**GTAGTTTATTACTCTTTAGCG
pbpF screening For	AGCTTGTACCAATGGGGAGC
pbpF screening Rev	TCAGTCCAAGAAAGCCTCCA
***ldtfm* deletion**	
ldtfm flanking Arm1_BamHI For	ATGCAT**GGATCC**TGTCTCACGCTTTGGCATTTA
ldtfm flanking Arm1_Sph1 Rev	ATGCAT**GCATGC**TCTTGTCATATCGACAACTCC
ldtfm flanking Arm2_Sph1 For	ATGCAT**GCATGC**GTCTTCTAACAAAAATAATAT
ldtfm flanking Arm2_BamHI rev	ATGCAT**GGATCC**TGGACGAACACCTATGTTCCA
ldtfm screening For	TACATTGGTGCACCTGCTGT
ldtfm screening Rev	GATGTATTAGAGGCGGGGGC
***ponA* deletion**	
ponA flanking Arm1_BamHI For	ATGCAT**GGATCC**TTCCCTTCATTCCAGTTATTA
ponA flanking Arm1_Xba1 Rev	ATGCAT**TCTAGA**AGTTTGTTCATTTGCCATTCT
ponA flanking Arm2_Xba1 For	ATGCAT**TCTAGA**GCAAATGACCATACACCATCCAGC
ponA flanking Arm2_BamHI rev	ATGCAT**GGATCC**TTCTTTAATTGCAAATAAATGTAT
ponA screening For	GGGACGCCCAAAAGGAAAAA
ponA screening Rev	AGATCACACATGTATATGCTGGA
***pbpZ* deletion**	
pbpZ flanking Arm1_Xba1 For	ATGCAT**TCTAGA**GCGTGTCGAATCCGATTGTTCCAA
pbpZ flanking Arm1_Sph1 Rev	ATGCAT**GCATGC**AAGGGTCACCTCAACTAATGGTTT
pbpZ flanking Arm2_Sph1 For	ATGCAT**GCATGC**GATGATTGATTTAATAGAATACAC
pbpZ flanking Arm2_Xba1 Rev	ATGCAT**TCTAGA**GCTAAGCAAGTGGACGGCGTGATC
pbpZ screening For	CATGCTGACGTGTGAGCCTA
pbpZ screening Rev	GCTGCATTTTGTTCACCACC
***pbpA* deletion**	
pbpA flanking Arm1_BamHI For	ATGCAT**GGATCC**AAAAATCGGTTTTCAAAGTCT
pbpA flanking Arm1_Sph1 Rev	ATGCAT**GCATGC**TTTTTTCATAAAATCTTTCAT
pbpA flanking Arm2_Sph1 For	ATGCAT**GCATGC**GCAAAATAAAAAAGAGGTCGTGAA
pbpA flanking Arm2_BamHI1 Rev	ATGCAT**GGATCC**ACAATTTTTGTCACAACCTTTTTT
pbpA screening For	GTAGTCGCATTAGCCGAGCT

pbpA screening Rev	TCTCGGTATGAAGTCAAATTTAAGCA
**For tetracycline marker**	
tetL For	[Phos]GACCGATGATGAAGAAAAGAATTTGAAAC
tetL Rev	[Phos]CTGTTATAAAAAAAGGATCAATTTTGAACTCTC
pbpA-linear For	GCAAAATAAAAAAGAGGTCGTGAA
pbpA-linear Rev	TTTTTTCATAAAATCTTTCAT
***ddcP* complementation**	
ddcP flanking Arm1_EcoRI For	ATGCAT**GAATTC**CGCCACCTGTCAGTTCAGATCCCA
ddcP flanking Arm1_EcoRI Rev	ATGCAT**GAATTC**TTGTTCCATGGCATATTGTTTCAT
***pst* transporter deletion**	
pst flanking Arm1_BamHI For	ATGCAT**GGATCC**CCTTGCAAAACCGTTTTTTGATGG
pst flanking Arm1_Sph1 Rev	ATGCAT**GCATGC**TAATTTAAGTTTTTTCATTAGAAA
pst flanking Arm2_Sph1 For	ATGCAT**GCATGC**TCAGGTCGATTTGGATAAGGAGGA
pst flanking Arm2_BamHI Rev	ATGCAT**GGATCC**TGAAGTAAGCACACAAATAAAAAA
pst screening For	TGTCCTTTTCTAACGGGGCC
pst screening Rev	CACGAACTGACTTGTGCACG
**Mutation confirmation**	
Transporter EFTG_01173 check for	GTGTTTCTGTTTCGGCTGGC
Transporter EFTG_01173 check rev	TTGTCATGTTGAGGCCTCCG
